# Optimizing Reliability of Real-Time Sonographic Examination and Scoring of Joint Synovitis in Rheumatoid Arthritis

**DOI:** 10.7759/cureus.31030

**Published:** 2022-11-02

**Authors:** Veena K Ranganath, Ami Ben-Artzi, Jenny Brook, Yosra Suliman, Astrid Floegel-Shetty, Thasia Woodworth, Mihaela Taylor, Laurie A Ramrattan, David Elashoff, Gurjit S Kaeley

**Affiliations:** 1 Department of Rheumatology, David Geffen School of Medicine, University of California Los Angeles, Los Angeles, USA; 2 Department of Rheumatology, Cedars Sinai Medical Center, Los Angeles, USA; 3 Department of Medicine Statistics Core, David Geffen School of Medicine, University of California Los Angeles, Los Angeles, USA; 4 College of Medicine, David Geffen School of Medicine, University of California Los Angeles, Los Angeles, USA; 5 Department of Rheumatology, University of Florida College of Medicine, Jacksonville, USA

**Keywords:** clinical trial, rheumatoid arthritis, outcome measures, synovitis, ultrasound

## Abstract

Objective: Musculoskeletal ultrasound real-time image acquisition and scoring are complex, and many factors affect reliability. Static image reliability does not guarantee real-time scoring. This study aimed to identify factors and solutions to improve real-time scoring reliability for the grey scale and power Doppler evaluation of synovitis. We also report on using a novel musculoskeletal ultrasound synovitis rule-based scoring atlas.

Methods: In four stages, we evaluated inter- and intra-reader reliability among three ultrasonographers (US1-3). Intra- and inter-reader reliability was calculated using weighted-kappa, intraclass correlation coefficient, and Spearman correlation. Reliability statistics were compared between stages using permutation tests to compute empirical distributions for differences in those statistics. At each stage, factors that diminished reliability were identified and addressed. After intensive reliability exercises, a RA MSUS atlas with in-depth scoring rules was generated to improve interpretive reliability.

Results: The three ultrasonographers had good to excellent intra-reader reliability for real-time acquisition scoring over 2432 views (weighted kappa 0.52-0.80, intraclass correlation coefficient 0.59-0.86, and Spearman correlation 0.64-0.86). Inter-reader reliability was good to excellent between US1/US2 and US1/US3 (weighted kappa 0.51-0.66, intraclass correlation coefficient 0.66-0.75, Spearman correlation 0.59-0.73). US1 achieved significant improvement in intra-reader reliability from stage 1 to stage 2 (*p*<0.05, weighted-kappa 0.63 to 0.80, intraclass correlation coefficient 0.71 to 0.86, Spearman 0.67 to 0.86) with use of the atlas.

Conclusion: This rheumatoid arthritis musculoskeletal ultrasound study addressed complex factors affecting musculoskeletal ultrasound acquisition-scoring reliability. Systematic identification and amelioration of many factors and using a novel rule-based scoring atlas significantly improved intra-reader reliability.

## Introduction

Tender and swollen joint counts are the cornerstone of disease activity measures in rheumatoid arthritis (RA) patients but are insensitive compared to imaging modalities such as musculoskeletal ultrasound (MSUS) [1]. MSUS provides a sensitive method for assessing synovitis by measuring greyscale synovial hypertrophy (GSUS) and synovial hypervascularization by power Doppler ultrasound (PDUS). PDUS synovitis predicts radiographic erosion progression [2-5] and rheumatoid arthritis flares [6,7], and PDUS may predict response to therapy as measured by clinical outcome [8-10]. Systematic literature reviews and expert panel recommendations by the American College of Rheumatology (ACR) and the European League Against Rheumatism (EULAR) have endorsed the use of MSUS to diagnose and monitor Rheumatoid Arthritis (RA) disease [11,12].

Despite these recommendations, the reliability of MSUS remains a significant concern. Known variability of acquisition and real-time scoring for GSUS and PDUS synovitis has hindered the full adoption of this method in randomized clinical trials [13-17]. Sources of variability are complex and include, but are not limited to sonographer experience, image acquisition, different brands of ultrasound machines, probes, settings, degree of deformities in subjects, and interpretive variability. Notable attempts in ameliorating acquisitional variability have included separating components of brightness (B)-mode imaging only to report synovial hypertrophy and not fluid [15-18]. Unfortunately, intra-observer reliability remained highly variable. Also, scoring on visible synovial hypertrophy assumes that joint fluid is inert and does not have inflammatory cytokines or cells, hence introducing a systematic bias to the scoring system [19]. Imaging atlases for radiographs and magnetic resonance imaging have been instrumental in creating consistent outcome measures in RA. However, few published RA MSUS atlases exist for use in clinical trials [16,20].

The objective of this study was to systematically identify factors and solutions to improve real-time scoring reliability for GSUS and PDUS before undertaking a bi-center prospective study of the effects of Tocilizumab in RA patients. This study reports on a multifaceted process to enhance acquisition reliability as defined by real-time scoring reliability [14,21]. First, we assessed still-image reliability at different stages to identify areas that required improvement. Second, we conducted an in-person acquisition and real-time scoring reliability exercise. Third, we iteratively and systematically identified and addressed critical factors of discrepancy. We created strict scoring rules for synovial areas to improve the scoring precision. A comprehensive novel reference atlas containing representative images and scoring rules was developed [20]. Fourth, we performed a second acquisition reliability exercise to test if there was an improvement in reliability. Fifth, we reviewed statistical methods and their nuances in examining reliability.

## Materials and methods

**Figure 1:**
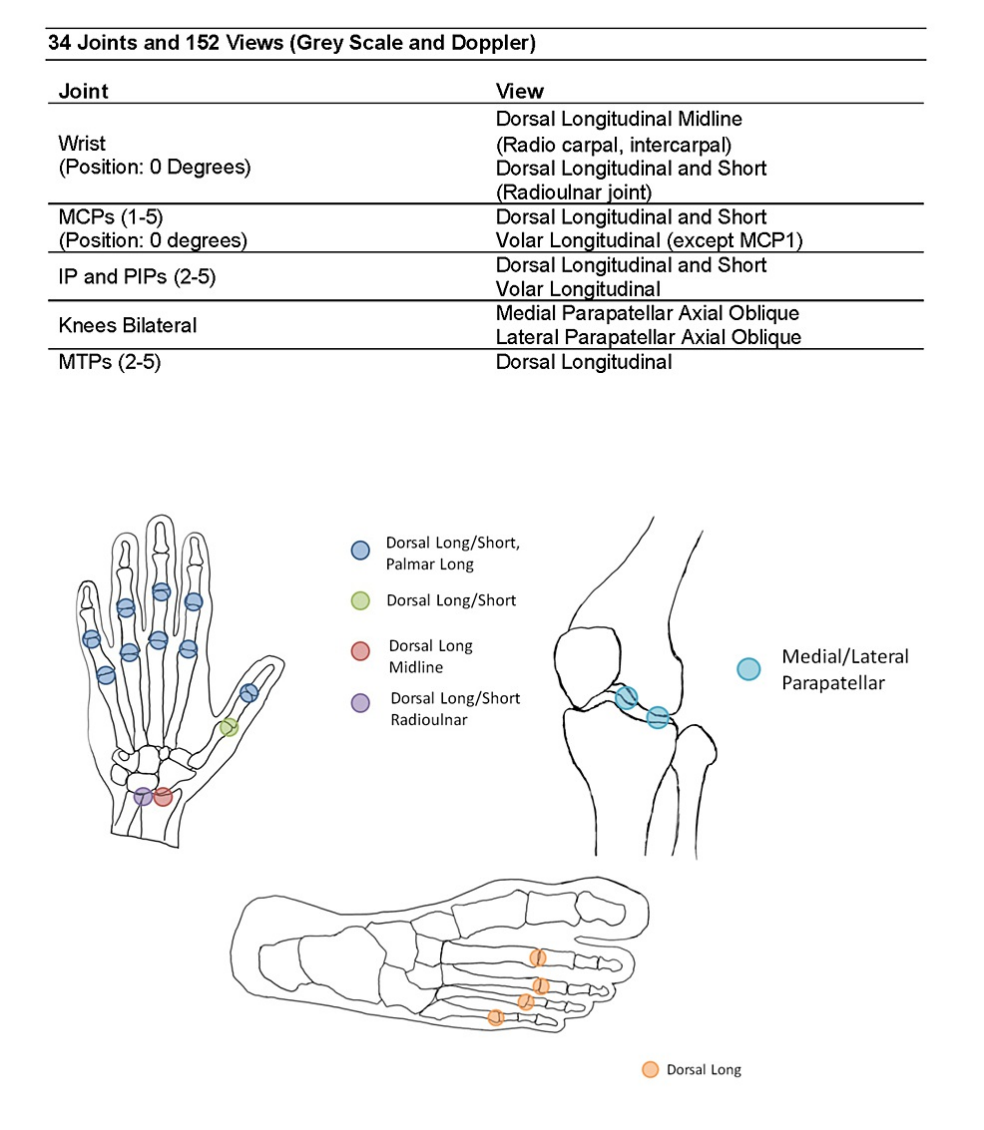
Views acquired across the joints MCP: Metacarpophalangeal joint, MTP: Metatarsophalangeal joint, IP: Interphalangeal joint, PIP: Proximal interphalangeal joint

The scanning protocol summarized in Figure 1 includes 34 joints: bilateral radio-carpal, intercarpal, radioulnar, metacarpophalangeal (MCP) 1-5, PIP 1-5, knees (parapatellar recesses), and metatarsophalangeal (MTP) 2-5. One hundred fifty-two views for both greyscale and power Doppler per patient (Figure 1). Since the suprapatellar recess of the knee is insensitive to Doppler examination, the parapatellar recesses were chosen. Currently, no consensus exists on which joints should be examined for optimal sensitivity to change. The joints and views were chosen based on joints commonly affected by RA and the feasibility of performing MSUS. This research activity was approved by the University of California, Los Angeles Institutional Review Board (IRB #12-001547) and registered with ClinicalTrials.gov (NCT01717859).

Patient positioning

The wrist and hand joints were examined in a neutral position. The knee parapatellar recesses are examined at 30 degrees flexion. The MTP joints were examined with the plantar aspects of the foot on the examination table. When joint contractures were present, the most relaxed position was assumed, and multiple acquisitions of the joint recesses were obtained to examine the joint fully. Gel layer standoff enabled the minimization of probe pressure.

Ultrasound machine and settings

Study sites utilized the Esaote My Lab Class C machine and a 12-18 megahertz (MHz) (LA-435) probe. US1 and US2 developed consensus grey scale settings for the Esaote My Lab Class C in a face-to-face meeting by analysis of RA patients with varying degrees of activity. Optimization of the greyscale image by the varying depth and B-Mode frequency was allowed. A red-yellow color map was used for the power Doppler map initially. For face to face-2 (F2F2) it was changed to a gradated blue color map due to the discovery that ultrasonographer 1 (US1) and ultrasonographer 2 (US2) were color-blind to red. The Doppler frequency was set at 8.3 MHz, the pulse repetition frequency (PRF) at 500 Hz, and the gain was just above the noise. The presets and software versions were harmonized across all study machines.

Scoring MSK-US RA atlas

Initial scoring on still images utilized a draft musculoskeletal ultrasound (MSK-US) RA atlas created after reviewing previously published atlases such as the Hammer et al. atlas [16]. Teleconferences to discuss discrepant scoring after each reliability exercise led to the creation of scoring rules incorporated into the atlas' future iterations. Revisions included strict rules for scoring depicted by MSUS images and joint diagrams [20].

Patients

Six patients who met ACR 1987 RA diagnostic criteria [22] and who had a clinical disease activity index (CDAI) >2.8 enrolled at the University of California, Los Angeles, Rheumatology outpatient clinics for the first acquisitional real-time scoring exercise (Face to Face 1 [F2F1]). Table 1 summarizes the components and scoring of the CDAI [23]. For the second acquisitional exercise (Face to Face 2 [F2F2]), four RA patients enrolled from the University of Florida Rheumatology College of Medicine, Jacksonville, outpatient clinics. Patients were instructed not to take non-steroidal anti-inflammatory drugs (NSAIDs) for 24 hours before the face to face (F2F) exercises. Patients were also instructed not to discuss their clinical disease activity with the ultrasonographers. The study was approved by the University of California, Los Angeles, institutional review board (IRB) and Western IRB (WIRB) and registered on clinicaltrials.gov (NCT01717859).

**Table 1:** Components of the Clinical Disease Activity Index (CDAI) and its interpretation.

CDAI Score	Rheumatoid Activity Level
0.0 – 2.8	Remission
2.9 – 10.0	Low Activity
10.1 – 22.0	Moderate Activity
22.1 – 76.0	High Activity
CDAI Score = SJC + TJC + PTG + PRG. SJC: Swollen joint count (0-28); TJC: Tender joint count (0-28); PTG: Patient global score (0-10); PRG: Provider global score (0-10)	

Sonographers' qualifications and participation in F2F1 and F2F2

Three sonographers (US1-3), each with at least seven years of experience (all Musculoskeletal Ultrasound Certified in Rheumatology certified by the American College of Rheumatology [RhMSUS]) participated in either F2F1 (US1 and US2) or F2F2 (US1 and US3).

Study design

**Figure 2:**
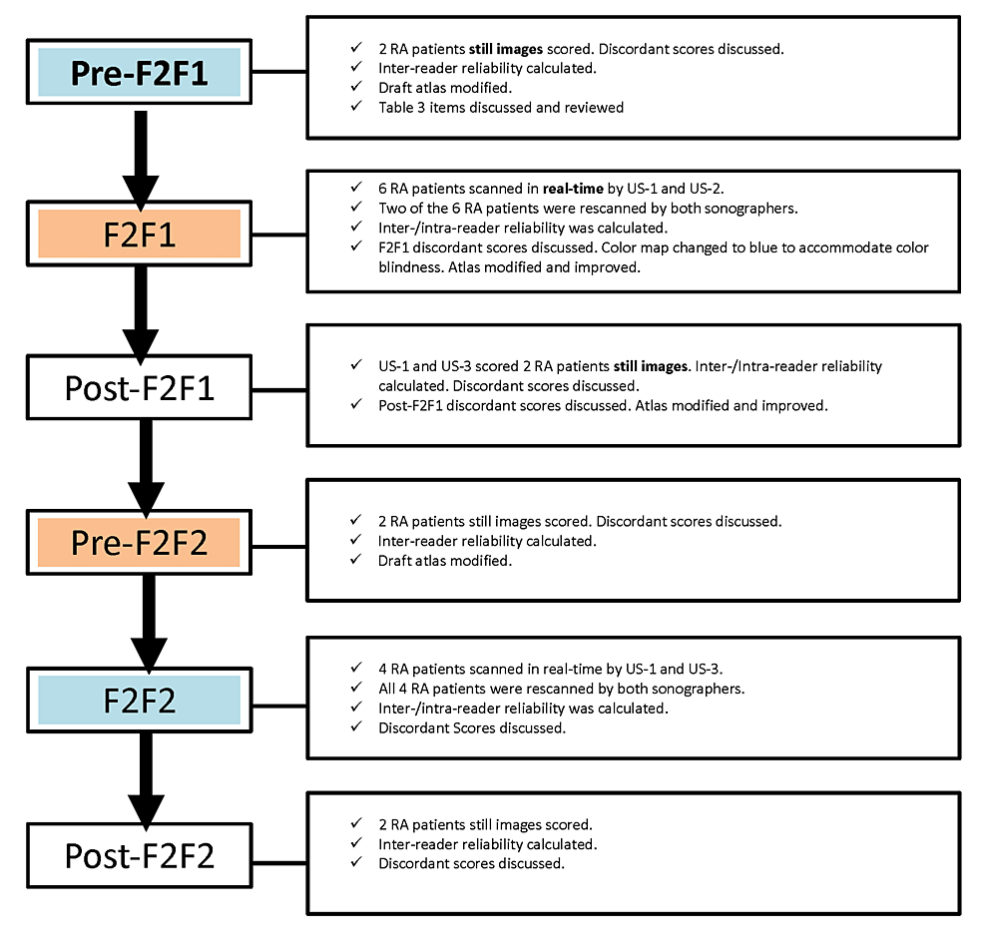
Flow of study procedures F2F1: Face to face meeting one, F2F2: Face to face meeting two, US-1: Ultrasonographer one, US-2: Ultrasonographer two, US-3: Ultrasonographer three, RA: Rheumatoid arthritis

The overall flow of study procedures is summarized in Figure 2. The two F2F meetings involving the three ultrasonographers occurred approximately five months apart to optimize the reliability of GSUS and PDUS acquisition and real-time scoring. GSUS and PDUS were scored on a semi-quantitative scale of 0-3, as suggested by MSUS guidelines [24]. Acquisitional reliability among three ultrasonographers was assessed in two in-person investigator meetings. 

Face to Face 1 in August 2014

**Figure 3:**
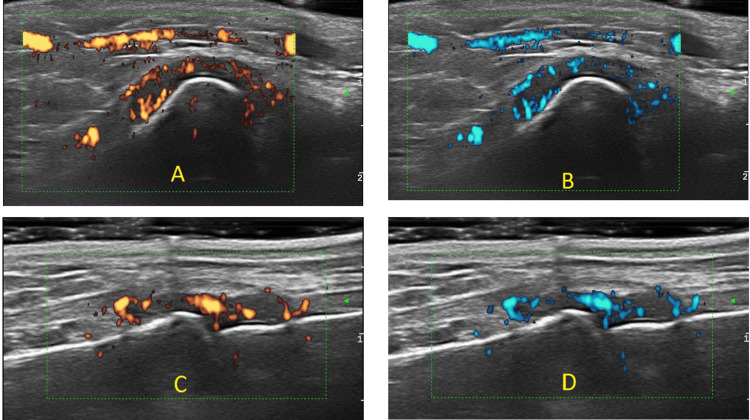
Color maps to accommodate red-green color blindness (A) Radioulnar long view with red-yellow color map, (B) radioulnar long view with gradated blue color map, (C) Metacarpophalangeal joint (MCP)1 long view with red-yellow color map, (D) MCP1 long view with gradated blue color map

**Figure 4:**
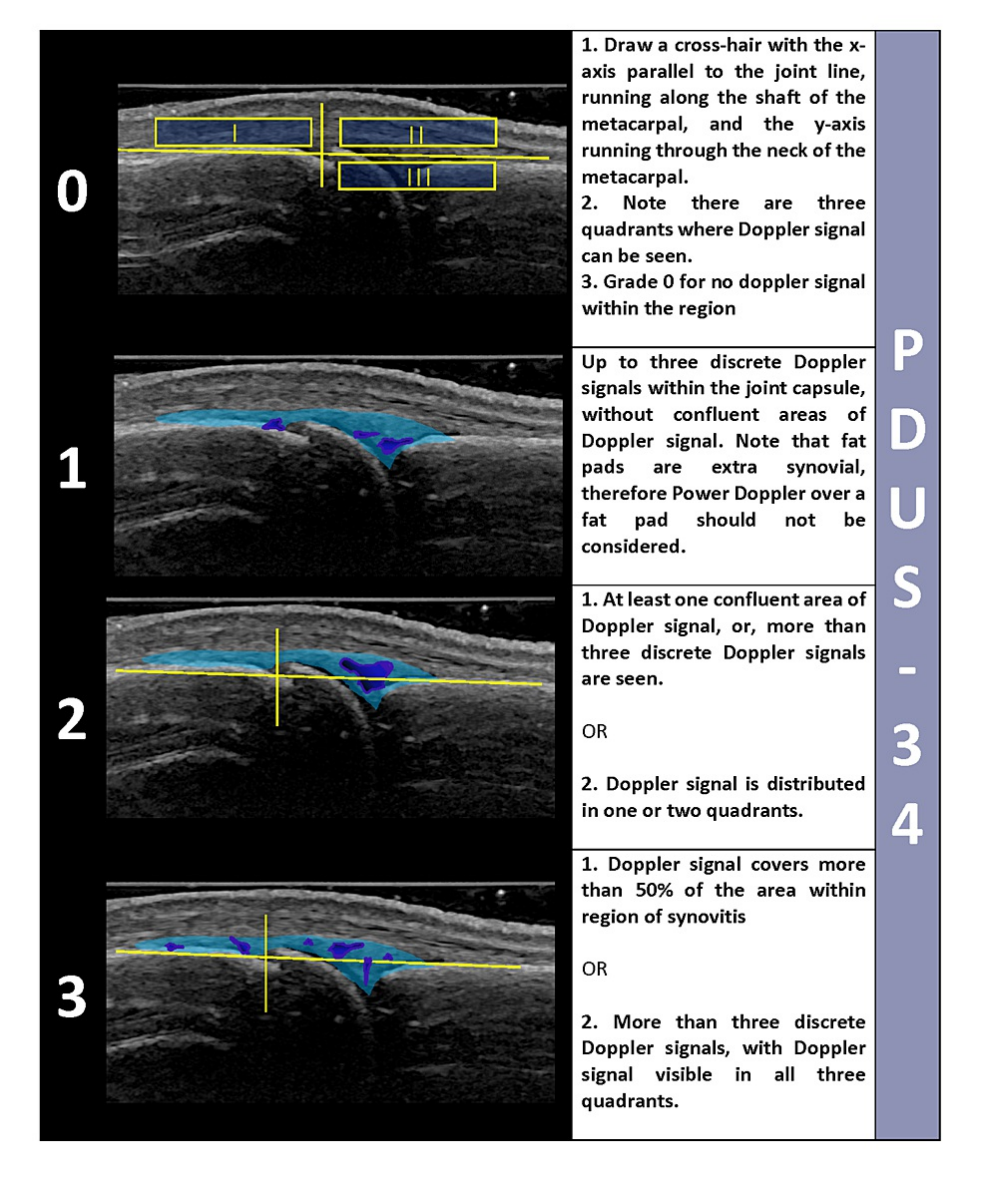
Example of scoring rules created to improve reliability This figure provides a sample of the 50% rule and the quadrant rule power Doppler scoring system for metacarpophalangeal joint (MCP) dorsal long view. The 50% rule traditionally differentiates between grade 2 and grade 3 scores. However, the 50% rule is sometimes difficult to apply in all situations; thus, the quadrant rule was developed to assist in these difficult-to-score circumstances. Power Doppler scores 0-3 are defined utilizing both rules.

For the Pre-F2F1, ultrasonographer 1 (US-1) and ultrasonographer 2 (US-2) independently scored still images of two RA patients. Based on inter-reader reliability scores, all three ultrasonographers discussed the most discrepant scores on the first day of the three-day F2F1 meeting. Scoring rules for both B-mode and Power Doppler were created to increase the consistency of rating. During the second day of the F2F1 meeting, six RA patients were scanned by US-1 and US-2. One hundred fifty-two views of 34-joints were scored in real-time per patient utilizing the draft atlas (for both GSUS and PDUS). Two of the six RA patients were scanned a second time the same day. Each sonographer acquired 1216 views during the day. Factors affecting discordance for the real-time acquisition scoring exercise were identified and addressed on the third day of the F2F1 meeting shown in Table 2. One significant change made after the F2F1 meeting was that the color map was changed from red-yellow to gradated blue to accommodate for color blindness (US-1 and US-2 were red-green color blind). An example of the color maps is shown in Figure 3. A Post-F2F1 reliability exercise of still images from two RA patients (Patient 1 and Patient 2 acquired by US-1 and Patient 1 and Patient 2 acquired by US-2 [total of 4 scans and 608 views]) was conducted approximately four weeks after the meeting. After F2F1, Post-F2F1, and teleconferences between ultrasonographers, the scoring atlas was modified, and for joints that were discrepant, scoring rules were created by consensus and included in the atlas. An example of the scoring atlas is given in Figure 4 [20].

**Table 2:** Variables affecting power Doppler ultrasound acquisition reliability

Factors to Improve Power Doppler Ultrasound Acquisition Reliability												
													Standardizing Environment and Equipment												
	Control Room Temperature and Lighting											
	Standardized Consensus for Power Doppler Settings and B-Mode											
	Familiarity with Ultrasound Machine											
Optimizing B-Mode and Doppler Acquisition												
	Standardized Layer of Gel											
	Focal point positioning											
	Optimize Depth and Frequency											
	Probe Positioning of Deformed Joints (optimizing recess views)											
	Coverage of All Recess Areas (may require two views for large recesses)											
	Strict Adherence to the Power Doppler Settings											
	Color Map Adjustment for Power Doppler to Accommodate for Color Blindness (present in 7% of males and 0.5% in females).											
Optimizing Scoring												
	Agreeing on Rules for Scoring in Compound Joints (such as the dorsal midline long wrist region)											
	Agreement of Synovitis Score Cutoffs											

Face to Face 2 in February 2015

Unexpectedly, US-2 could not continue working on the multi-center ultrasound RA study. Thus, US-3 served as the ultrasonographer, and it was determined that a second F2F meeting was necessary to establish acquisition real-time scoring reliability between US-1 and US-3. The novel RA MSUS atlas with in-depth scoring rules was used. Before F2F2 (Pre-F2F2), US-1 and US-3 scored 304 still images from two patients, and reliability was calculated, similar to the exercise conducted pre-F2F1. Discrepancies were discussed between US-1 and US-3 via teleconferences. F2F2 was a two-day investigator meeting. US-1 and US-3 scanned a total of four RA patients, and then all four patients were rescanned. Each sonographer acquired 1216 views during the day. The inter-and intra-reader reliability for acquisition and real-time scoring was calculated, and a post-F2F2 reliability exercise analyzing still images was performed similarly to post-F2F1.

Statistics

The initial analysis summarized each joint's B-Mode synovial hypertrophy and PDUS by computing the maximum score across the set of views for that joint. Thus, these summary scores were also on an ordinal scale from 0 to 3. Intra-reader reliability statistics were calculated at the joint level using the two scores obtained by each reader for each patient's joint. Inter-reader reliability statistics were calculated by comparing the individual scores for each patient's joint obtained by each pair of readers during their first reading. The measures of agreement were percent agreement, weighted kappa coefficient, Spearman correlation coefficient, and intra-class correlation (ICC) [25]. The weighted kappa was calculated using weights from the column scores (Fleiss-Cohen weights) [26]. We also estimated the bias between the readers by calculating the difference between the average PDUS joint value for each reader. Additionally, the prevalence of zeros was estimated by the overall proportion of zero scores across all joint PDUS values for each reader. All agreement statistics were compared between F2F1 and F2F2 using permutation simulations. With each iteration of the simulation, we randomly permuted the joint level PDUS scores for both readers across the groups (F2F1 vs. F2F2). Next, we computed the overall agreement measures separately for each group using all joint level data assigned to that group. Finally, we computed the difference in the agreement measures between groups. The simulation used 1000 iterations. This process allowed us to compute empirical distributions for differences in each agreement statistic. The p values for the permutation tests were based on the proportion of permutations, resulting in larger differences between F2F1 and F2F2 than the observed difference. All analyses were done using SAS 9.4.

## Results

Patient characteristics

The six RA patients who participated in F2F1 had a mean age of 61 years (standard deviation (SD) 13.5), a mean disease duration of 7.47 years (SD 8.37), and a mean CDAI of 21 (SD 10.7) (CDAI > 20 denotes severe RA disease activity). The four RA patients who participated in F2F2 had a mean age of 62.3 years old (SD 1.70), a mean disease duration of 16 years (SD 4.2), and a mean CDAI of 34.5 (SD 9.2). Nine out of the 10 patients were female. A total of 1216 PDUS and GSUS views were obtained per sonographer at F2F1 (six patients and two rescanned) and at F2F2 (four patients and all rescanned).

Inter-reader reliability for still-image scoring (pre-F2F1, post-F2F1, pre-F2F2, and post-F2F2)

Overall, still-image inter-reader reliability for GSUS and PDUS scoring after the in-person meetings resulted in higher weighted Kappas compared to the still-image inter-reader reliability performed before the in-person session (GSUS 0.25-0.56 prior and 0.72-0.74 after; PDUS 0.60-0.77 prior and 0.77-0.84 after). Percent agreement, ICC, and Spearman correlation followed similar trends. Still image GSUS and PDUS reliability details are shown in Tables 3, 4. 

**Table 3:** Still image GSUS ultrasound scoring reliability during pre-/post- F2F1 and F2F2 ICC: Intraclass correlation coefficient; F2F: Face-to-face; US: Ultrasonographer

		% Agreement	Weighted Kappa	ICC	Spearman correlation
						Inter-reader pre-F2F1					
	US-1/US-2	59	0.25	0.36	0.39
Inter-reader post-F2F1					
	US-1/US-2	75	0.72	0.74	0.8
Inter-reader pre-F2F2					
	US-1/US-3	63	0.56	0.66	0.64
Inter-reader post-F2F2					
	US-1/US-3	69	0.74	0.81	0.84

**Table 4:** Still image PDUS scoring reliability during pre-/post- F2F1 and F2F2 ICC: Intraclass correlation coefficient; F2F: Face-to-face; US: Ultrasonographer

		% Agreement	Weighted Kappa	ICC	Spearman correlation
						Inter-reader pre-F2F1					
	US-1/US-2	62	0.6	0.75	0.79
Inter-reader post-F2F1					
	US-1/US-2	82	0.77	0.75	0.83
Inter-reader pre-F2F2					
	US-1/US-3	85	0.77	0.84	0.82
Inter-reader post-F2F2					
	US-1/US-3	81	0.84	0.82	0.91

Intra- and inter-reader reliability for acquisition and real-time image scoring (F2F1 and F2F2)

Percent agreement, weighted kappa, ICC, and Spearman correlation of GSUS and PDUS were calculated for real-time scoring. GSUS real-time acquisition intra-reader reliability scoring improved from F2F1 (0.66-0.61) to F2F2 (0.76-0.63) for weighted kappa, as shown in Table 5. Of note US1’s real-time intra-reader weighted kappa improved from 0.66 to 0.76. GSUS inter-reader weighted kappa improved from 0.4 to 0.56 (p=0.01) from F2F1 TO F2F2, respectively.

**Table 5:** Acquisition and real-time GSUS scoring reliability ICC: Intraclass correlation coefficient; F2F: Face-to-face; US: Ultrasonographer

Intra-reader					
		% Agreement	Weighted	ICC	Spearman
			Kappa		correlation
F2F-1	US-1 GK	77	0.66	0.69	0.68
	US-2 AB	78	0.61	0.72	0.69
F2F-2	US-1 GK	76	0.76	0.81	0.89
	US-3 VR	63	0.63	0.76	0.86
	Difference	1	-0.1	-0.12	-0.21
	US-1				
	p-value	0.43	0.08	0.21	0.005
Inter-reader					
		% Agreement	Weighted	ICC	Spearman
			Kappa		Correlation
F2F-1	US-1/US-2	55	0.4	0.5	0.43
F2F-2	US-1/US-3	56	0.56	0.74	0.69
	Difference	-1	-0.16	-0.14	-0.26
	p-value	0.43	0.01	0.09	0.0008

PDUS acquisition intra-reader weighted kappa improved from F2F1 (0.55-0.61) to F2F2 (0.62-0.76). The intra-reader PDUS reliability of US-1 improved from F2F1 to F2F2 for weighted kappa (p=0.1) and Spearman correlation (p<0.05) (Table 6). However, there was a trend toward worsening percent agreement for US-1 from F2F1 to F2F2. Inter-reader reliability also improved from 0.50 to 0.64 due to between-meeting consensus activities. A statistically significant improvement of power Doppler inter-reader reliability between F2F1 to F2F2 was seen for weighted kappa (p=0.03). For both GSUS and PDUS, percent agreement, ICC, and Spearman correlation followed similar trends to the weighted kappa trends, as shown in Tables 4, 6.

**Table 6:** Acquisition and real-time PDUS scoring reliability ICC: Intraclass correlation coefficient; F2F: Face-to-face; US: Ultrasonographer

Intra-reader					
		% Agreement	Weighted	ICC	Spearman
			Kappa		Correlation
F2F-1	US-1 GK	93	0.67	0.71	0. 73
	US-2 AB	85	0.55	0.59	0. 63
F2F-2	US-1 GK	79	0. 76	0.86	0.96
	US-3 VR	66	0. 62	0.8	0.89
	Difference	14	-0.09	-0.15	-0.23
	US-1				
	p-value	0.001	0.1	0.17	0.002
Inter-reader					
		% Agreement	Weighted	ICC	Spearman
			Kappa		correlation
F2F-1	US-1/US-2	76	0.5	0.66	0.56
F2F-2	US-1/US-3	65	0.64	0.74	0.72
	Difference	11	-0.14	-0.08	-0.16
	p-value	0.01	0.03	0.22	0.02

There was a significant worsening in inter-reader percent agreement from F2F1 to F2F2 (p=0.01). As stated above, the average CDAI was lower for F2F1 compared to F2F2 (21 vs. 34.5). The average prevalence of PDUS zeros in F2F1 was 82% for US-1 and US-2, whereas the prevalence of zeros in F2F2 was 46% for US-1 and US-3. For GSUS, the average prevalence in F2F1 was 55% for US-1 and US-2, and the prevalence of zeros in F2F2 was 27% for US-1 and US-3.

## Discussion

Demonstration of excellent reliability in outcome measures is necessary before conducting multicenter randomized controlled clinical trials. Imaging outcome measures in RA with proven track records, such as radiographs (Sharp scores) and RA magnetic resonance imaging (MRI) scoring (RAMRIS), have utilized atlases to demonstrate high-reliability rates. There are at least two sources of variability for inter-and intra-reader reliability in MSUS data: 1) acquisition of MSUS images and 2) MSUS image scoring. During the iterative process of the study, we identified factors that affect the acquisitional and scoring reliability of MSUS images from RA patients, which are summarized in Table 2. Addressing these factors and using a novel detailed, in-depth MSUS atlas for PDUS scoring may mitigate sources of variability in a clinical trial utilizing joint ultrasound as an outcome measure. Our study demonstrates that the real-time intra-reader acquisition reliability of PDUS in RA patients can be significantly improved after using this atlas. While reliability for still-image MSUS scoring is quite good (and also can be improved), we and others have established that it is not sufficient to assure good to excellent reliability for real-time acquisitional scoring across an extensive set of joints [18]. In addition to the interpretation of images, the acquisition must control other factors summarized in Table 2 that may affect PDUS, and standardization must be carefully followed. Here, we report for the first time that good to excellent real-time acquisition scoring reliability is achievable in real-time detailed scanning of an extensive set of joints. Importantly, these results assure that greyscale and PDUS synovitis scoring can be dependably utilized in RA multicenter clinical trials.

There are RA clinical trial designs that demand real-time scoring for enrollment or treatment decisions (i.e., treat to target or flare) [27-30]. Incorporating ultrasound-based therapeutic decision-making in rheumatological clinical practices would also necessitate the real-time acquisition of images to assess synovitis. Real-time PDUS scoring is advantageous due to the ease of collecting and entering data. This would also most accurately reflect the daily practice patterns of rheumatologists.

Our study is unique in that reliability exercises were done at multiple stages (with a total of 1216 still images and 2432 real-time acquisition/scoring views). The problems identified were used to drive iterative systematic adjustments to the scanning techniques and interpretation of images. Furthermore, extensive detailed scanning generated a robust number of views across various joints. Factors that maximized sensitivity included optimizing the B-Mode image collection, using a gel layer for Doppler scanning, changing the Doppler color map, and attempting to bring joints into a neutral position if joint deformities were present. Although these factors had all previously been reported to influence sensitivity, reminders of good sonographic habits are essential - no matter how experienced the sonographer may be. The iterative process, including changing the color Doppler map, likely contributed to improved reliability of US1 from F2F1 to F2F2. The atlas created during this study expanded on prior atlases and included explicit rules for grading both greyscale and Doppler images. In addition, the atlas addresses the difficulty of deciding between grade 2 and 3 Doppler signals by using a novel quadrant method. During the study period, we also encountered a drop-out of one of the sonographers; this might occur during multicenter RA studies. This allowed us to demonstrate that we could recreate the training process with a backup sonographer and in patients with more severe diseases.

Limitations of our study relate to the feasibility of recreating multiple F2F training sessions to improve acquisitional reliability, although this would be a worthwhile investment for a large trial. It may also be problematic in large multicenter MSUS studies to conduct a real-time acquisitional reliability exercise as we did here. One way to mitigate this issue would be to have a central MSUS scorer review scans for quality assurance before the inclusion of an MSUS site. This ultrasonographer would assess for factors known to improve PDUS reliability. In addition, MSUS of 34 joints may not be feasible in some RA clinical trials, and a consensus is needed regarding the joints scanned and methods for composite scoring.

Our results also establish that the statistical method used to examine PDUS reliability in rheumatoid arthritis requires careful consideration. Each of these reliability measures has strengths and limitations that are important to note. Differing disease activity levels found in RA patients can significantly affect the reliability assessment of percent agreement. Understandably, RA patients with minimal disease activity by CDAI will have more MSUS images with PDUS scores of zero. More images with zero scores led to a higher percent agreement for intra- and inter-reader reliability. In this study, 82% of F2F1 scores were classified as zeros, whereas F2F2 had 47% zero scores (also reflected in differences in the mean CDAI scores). The percent agreement was higher in the F2F1 phase. For this reason, we do not advocate the sole use of percent agreement for assessing reliability.

Similarly, unweighted kappa statistics may not be ideal for evaluating inter- and intra-reader agreement with semi-quantitative measures such as PDUS, since discordance in the scoring of 0 vs. 3 would be treated equivalently to 2 vs. 3. In contrast, the weighted kappa considers the magnitude of the agreement or disagreement and better corrects for the fact that a discrepancy of 0 vs. 3 should not be viewed similarly to a discrepancy of 2 vs. 3. It is important also to note that kappa statistics are affected by both the overall prevalence (proportion of non-zero PDUS scores) and bias between raters [31]. 

ICC evaluates agreement with quantitative measures and may not be ideal for evaluating reliability for the ordinal scale of the individual scores of views or individual joint scores; however, it is a good measure for overall patient PDUS scores. A limitation of the ICC is that it is affected by the population variability or skewness in the outcome, and differences between ICCs can result from differences in the variability in the populations assessed.

Although the Spearman correlation is not a traditional measure of agreement, and we do not specifically advocate for its utilization, Spearman correlations help examine relationships within and between ordinal measures. In contrast to ICCs, the use of Spearman correlations to evaluate reliability at the joint level or view level is appropriate. Spearman does adjust for bias; therefore, it is important to evaluate bias for systematic differences in the magnitude of the score. No one statistical reliability measure is ideal since each measure has drawbacks. Nonetheless, our study demonstrates that all three measures show improvement in intra-rater agreement due to our F2F process.

## Conclusions

In conclusion, we have systematically optimized the reliability of real-time MSUS scoring across an extensive set of joints by creating a novel atlas with strict scoring rules to enable a multicenter MSUS rheumatoid arthritis study. Our study provides a comprehensive method for optimizing still image reliability and real-time data scoring of acquired images. Excellent still-image scoring reliability was insufficient to ensure real-time scoring accuracy. The reliability exercises should be conducted in patients with severe disease activity rather than in RA patients with low disease activity due to fewer zero scores, as it is easier to attain good reliability with zero scores than higher scores. While used commonly in RA studies, percentage agreement is not a good measure of reliability and overestimates agreement in RA patients with low disease activity. These results, for the first time, suggest that acquisition and real-time scoring reliability can be significantly improved holistically across a heterogeneous set of joints. We have established a model for attaining excellent MSUS reliability for future RA multicenter trials.
